# Unveiling Spin Selective Mechanism toward Very Large Spin Signals in Black Phosphorus 2D Semiconductor Spintronics Devices

**DOI:** 10.1021/acsami.5c07758

**Published:** 2025-08-28

**Authors:** Marta Galbiati, Simon M.-M. Dubois, Hao Wei, Victor Zatko, Julian Peiro, Florian Godel, Regina Galceran, Pierre Brus, Frédéric Fossard, Jean Sébastien Mérot, Etienne Carré, Etienne Gaufrès, Annick Loiseau, Jean-Christophe Charlier, Frédéric Petroff, Marie-Blandine Martin, Bruno Dlubak, Pierre Seneor

**Affiliations:** † Laboratoire Albert Fert, CNRS, Thales, Université Paris-Saclay, 91767 Palaiseau, France; ‡ Institute of Condensed Matter and Nanosciences (IMCN), Université Catholique de Louvain, B-1348 Louvain-la-Neuve, Belgium; § 84360 Thales Research and Technology, 91767 Palaiseau, France; ∥ Laboratoire d’Étude des Microstructures, UMR 104 CNRS-Onera, 92322 Châtillon, France; ⊥ Instituto de Ciencia Molecular (ICMol), Universitat de València, Paterna, Valencia 46980, Spain

**Keywords:** black phosphorus, spintronics, magnetic tunnel junctions, 2D materials, magnetoresistance, spin filtering, spin valves, spintronic devices

## Abstract

Discovering an efficient spintronic semiconductor workhorse with dual host capabilities as a channel and spin valve barrier remains one of the most elusive endeavors toward the development of spin-logic circuits. Graphene paved the way for two-dimensional (2D) materials, yet engineering a controlled band gap in it remains a challenge. Black phosphorus (BP) was recently unveiled as a potential candidate in the realm of 2D semiconductors, with carrier mobilities among the largest reported for a 2D material and a low spin–orbit coupling reminiscent of graphene. Although promising spin transport properties have already been reported, their potential for tunneling and spin injection remains uncharted. Here, we unveil an unknown spin transport mechanism spin-split in k-space and report on corresponding high magnetoresistance spin signals up to 500% in BP based spin valves. Those findings are analyzed and discussed in light of a first-principles theoretical investigation showing BP’s potential for spin filtering beyond its expected role of spin transport channel. This strongly supports BP’s vision as an outstanding platform for spintronics, as it could become a versatile workhorse yet unavailable with any other semiconductor.

## Graphical abstract

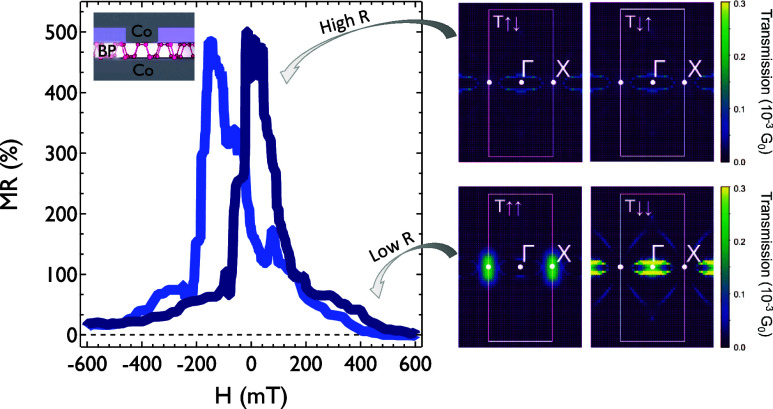

## Introduction

Spintronics is at the heart of widely distributed applications such as hard drive read heads and magnetoresistive random-access memories (MRAMs) and is seen as a solid candidate for beyond complementary metal–oxide–semiconductor (CMOS) architectures including spin-logic, neuromorphic, stochastic, and quantum technological layers.^1–7^ The spintronics community is hence actively looking for material platforms compatible with spin transport and manipulation. In this direction, 2D materials have been attracting a strong interest in the last years thanks to the rich variety of available layered materials which offer appealing properties for spintronics applications, such as atomically defined interfaces, free of defect barriers, spin filtering, perpendicular anisotropy, and spin–orbit torque modulation.^8–14^ Promising results have been obtained using graphene^15–17^ and hexagonal boron nitride (h-BN),^18^ directly integrated in 2D-magnetic tunnel junctions (MTJs) by chemical vapor deposition (CVD). In the same vein, 2D semiconductors such as transition metal dichalcogenides (TMDCs) have now been explored for MTJs,^19–29^ and while their difficult integration has so far limited their exploration and performances, the first sizable spin signals have begun to be measured^27^ with hints of band structure spin-filtering effects.^28^ Still, a performant spintronic semiconductor host able to work as a spin channel and spin valve barrier is yet unavailable and remains one of the most fundamental and elusive questions toward the development of spin-logic circuits.

To this aim, black phosphorus (BP) has sparked great hopes for spintronics applications. Indeed, its large carrier mobilities^30–34^ and expected intrinsically low spin–orbit coupling^35^ make it especially appealing for long distance spin transport. In addition, its tunable bandgap (from 0.3 eV for the bulk to around 1.9 eV in the monolayer)^34,36–38^ naturally bridges the range from zero bandgap graphene to TMDCs.^2,39,40^ Hence, BP could become a semiconductor analogous to graphene for spin transport, while offering the additional advantage of gate tunability. Interestingly, theoretical studies have already predicted relatively large MR signals in BP-based spin valves, unveiling its potential for spin injection.^41–44^ Nevertheless, until now only few pioneering results have been reported in the literature on lateral or vertical spin transport.^45,46^ Indeed, integrating BP in spin valve devices has proven particularly challenging due to its rapid photo-oxidation in ambient conditions, which drastically compromises its crystalline structure and transport properties.^47–49^

In this work, we report on high MR spin signals from 90% reaching up to 500% in Co/BP/Co based spin valve junctions. Those findings correspond to a large improvement—from 1 to 3 orders of magnitude—in terms of spin signal compared to previous results reported in the literature on 2D semiconductors^19–29^ or BP^46^ based MTJs. We discuss how these results arise from a selective transport mechanism proper to BP spin-split in the reciprocal space, as supported by first-principles theoretical investigations. We thus present BP to simultaneously play the role of a high potential injection/tunnel barrier in addition to its already known efficiency as a spin transport channel.

## Results and Discussion

In our experiments, the successful integration of mechanically exfoliated BP flakes in spin valves relies on a fabrication protocol which employs passivation layers grown by atomic layer deposition (ALD)^50^ to preserve the integrity of both the ferromagnet and the 2D flakes to take full advantage of BP toward large spin signals (Figure 1a), to which we dedicate a special effort. First, a Co bottom electrode is deposited by magnetron sputtering in an adapted spintronics growth cluster. Using a dedicated vacuum suitcase, the Co sample is directly transferred to a glovebox avoiding any surface oxidation. There, BP flakes are deposited on the Co surface through mechanical exfoliation. The sample is then transferred from the glovebox to an ALD growth chamber using the vacuum suitcase and a passivation layer is grown following our already established passivation processes.^16,51^ Thus, during the whole process, air exposure of BP is avoided.

**Figure 1:**
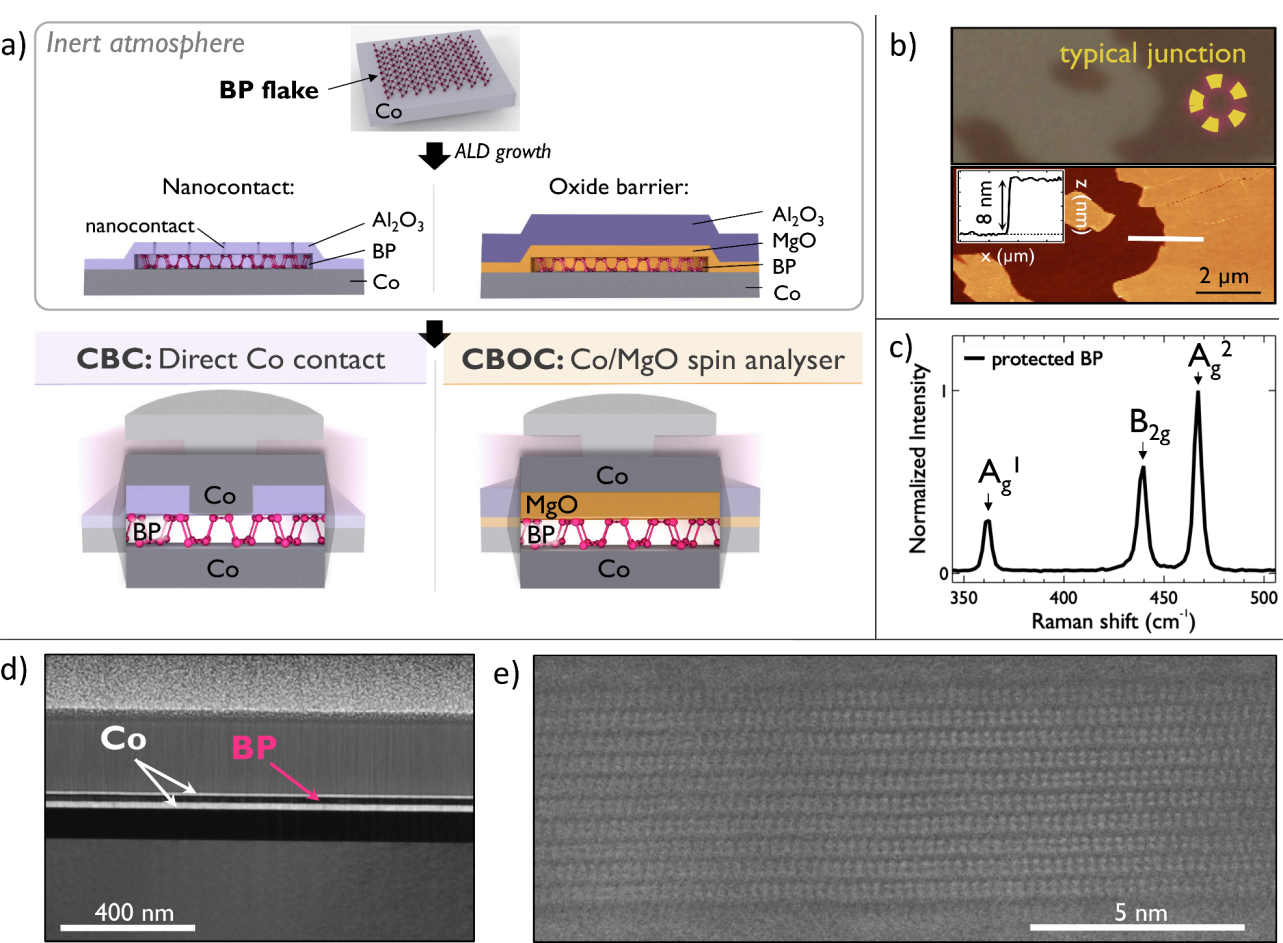
(a) Schematic of the black phosphorus (BP) device fabrication process to preserve and integrate the delicate Co/BP interface in complete spin valve junctions through their protection with ALD grown passivation layers. The schematic of the final Co/BP/Co (Sample CBC) and Co/BP/MgO/Co (Sample CBOC) devices are displayed at the bottom. (b) Top: optical microscope image of a BP flake deposited over the Co bottom electrode and protected with the Al_2_O_3_ barrier (Sample CBC). Bottom: AFM tapping image of the same flake and (inset) its profile. (c) Raman spectrum of the protected BP flake after days of air exposure. Peaks of BP are still well visible, further highlighting the preservation of the BP layer. (d) HAADF SEM image of a cross-sectional lamella with a thickness of 50 nm of our junctions. (e) STEM image of a BP multilayer after passivation with our method. The measured interlayer spacing of 0.53 nm is in line with expected BP crystal parameters. The high quality of the layers visible at the interfaces validates the effectiveness of our passivation method to preserve 2D layer quality in the fabrication process.

In the following section, we explore two kinds of samples. First, Co/BP/Co junctions (Sample CBC) are measured to investigate BP’s potential for spintronics. Due to the instability of BP in ambient conditions, achieving a high-quality direct contact for CBC devices is highly challenging. To address this, we developed a special fabrication process in which a protective Al_2_O_3_ layer is grown by ALD at its wettability limit (as reported in ref 16). This approach both protects the 2D layer and enables the formation of a direct metal contact over an extremely small area, hence creating a nanocontact.

Then, we also fabricated hybrid Co/BP/MgO/Co junctions (Sample CBOC), where a (∼2 nm) MgO layer grown by ALD is inserted so that the top MgO/Co is used as spin analyzer of the Co/BP interface while protecting BP to maintain its highest quality.^50–52^ To further protect the MgO from interacting with the ambient atmosphere and the following fabrication steps, a 10 nm Al_2_O_3_ layer is consecutively grown in situ on top of the MgO as reported in previous work and removed just over the contact junction during the lithography process.^51^

Once the ALD process is completed, BP flakes are first spotted by using an optical microscope. An example of a BP flake deposited on the Co bottom electrode and further passivated and exposed to ambient conditions is shown in Figure 1b. No difference is observed on flake integrity between samples of both types CBC and CBOC. From optical contrast it is possible to gain a first idea of flakes thickness as their visibility is not hampered by the ultrathin passivation barrier. Atomic force microscopy (AFM) is subsequently used to determine flake thickness. Figure 1b shows the topography image of a typical flake with a thickness of ∼8 nm and a roughness with a root-mean-square (RMS) parameter of ∼0.2 nm, which proves the high quality of the flake surface. No signs of degradation are observed, as expected.^50,51,53^ BP’s structural quality preservation over time is also confirmed by Raman spectroscopy. Figure 1c presents the Raman spectrum measured over the protected flake after a few days in ambient air using an excitation laser of 514 nm. The vibrational modes A_g_^1^, B_2g_, and A_g_^2^ characteristic of BP are still intense and visible at 361, 438, and 466 cm^–1^, respectively, in line with results reported in the literature.^47,53^ This confirms the maintained quality of the flakes, as damaged BP exhibits a complete decrease of its Raman signature peaks intensity.^53^ To further validate the preservation of 2D layer quality after our passivation process, we present a high-angle annular dark field scanning transmission electron microscopy (HAADF-SEM) image of a cross-sectional lamella of our junctions (Figure 1d), clearly showing that the layers remain undamaged. The high quality of BP is also evidenced by transmission electron microscopy (TEM) images, which reveal well-defined interfaces after passivation (Figure 1e). These results confirm the effectiveness of our passivation method in maintaining high-quality 2D layers within our junctions.

Once flakes have been selected and characterized, we proceeded to junction fabrication through a lithography process, resulting in contacts of 1–2 μm diameter size, finally completing the device with the deposition of the top Co (15 nm)/Au (80 nm) electrode by sputtering. The schematics of the final Co/BP/Co and Co/BP/MgO/Co junctions are represented in Figure 1a.

For transport characterization we first focus on the BP only Co/BP/Co nanojunction (Sample CBC) and we report the case of a contacted flake with a thickness of about 6 nm measured at low temperature to ensure the best possible defined magnetic states and reduce thermal contributions. Strikingly, an extremely large MR signal up to 500% is observed in our best device (Figure 2). Here the switching fields are controlled by the ultrasmall local geometry, dominating the micromagnetic behavior. This measurement remarkably represents one of the highest MR values reported so far in 2D semiconductor-based spin valves, with a gain of 3 orders of magnitude compared to signals previously reported for BP-based MTJs in the literature.^46^ Applying Julliere’s model, we can naively estimate the spin polarization between Co and BP by considering, in a first approximation, that both interfaces are equivalent: MR = 2*P*_Co/BP_^2^/(1 – *P*_Co/BP_^2^). This results in a spin polarization for Co/BP of *P*_Co/BP_ ≈ 85%.

**Figure 2:**
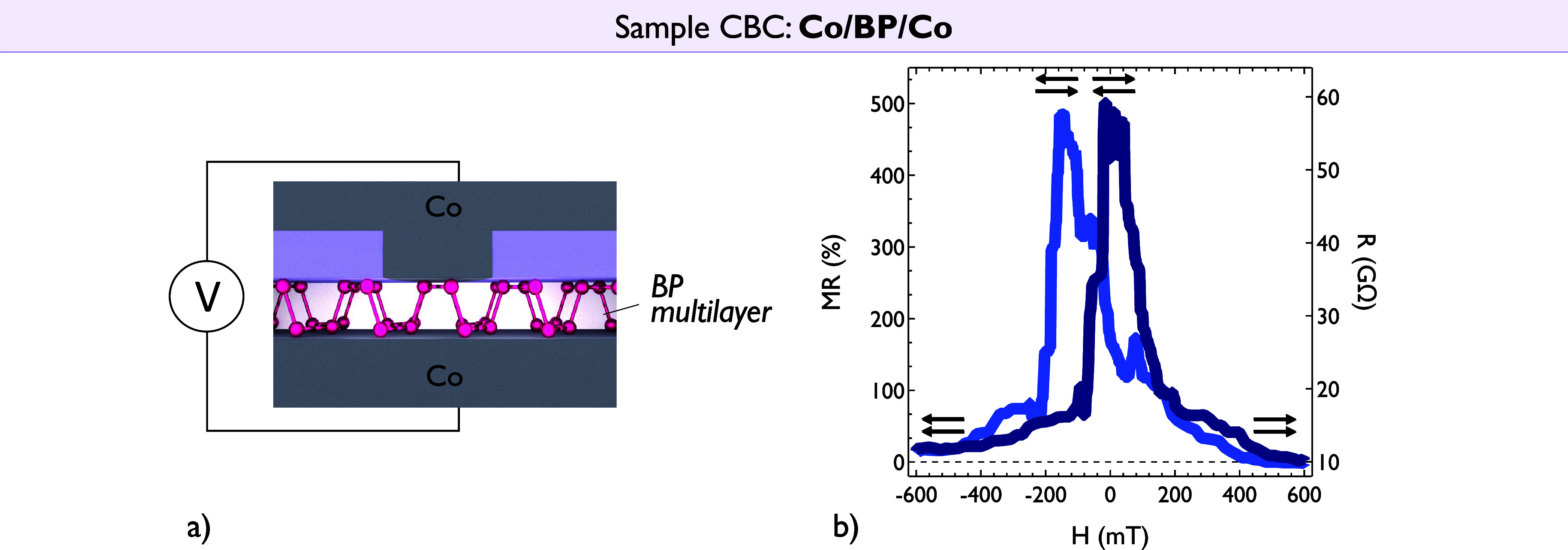
Magneto-transport measurements in a Co/BP/Co spin valve (Sample CBC). (a) Schematic of the Co/BP/Co nanojunction. (b) MR signal (left axis) of 500% (with 15% error margin) and the corresponding resistance R signal (right axis) measured at 2 K in the Co/BP/Co junction. The spin signal reached in this symmetrical junction exceeds the one obtained in the case of the Co/MgO analyzer in Figure 3, supporting the strong spin filtering effects arising in the Co/BP/Co structure.

The measured MR spin signal and corresponding extracted spin polarization are particularly large for semiconductor-based MTJs. We thus introduce another type of device, hybrid Co/BP/MgO/Co junction (Sample CBOC), with a MgO/Co spin analyzer used to specifically probe the Co/BP interface and further evaluate its spin polarization. Figure 3 shows magneto-transport measurements performed at a low temperature on this device. The *IV* curve shows a nonlinear behavior, and a zero-bias anomaly^54^ compatible with tunnel transport is observed in the d*I*/d*V* curve collected using an alternating current + direct current (AC+DC) lock-in system. A large positive MR signal up to 90% is measured. Following Julliere’s formula MR = 2*P*_Co/BP_ *P*_Co*/*MgO_/(1 – *P*_Co*/*BP_ *P*_Co*/*MgO_), we conservatively consider a spin polarization in our reference analyzer as *P*_Co/MgO_ ≈ 40%. Indeed, our MgO is not crystalline and optimized through annealing and hence with a limited spin polarization usually below this value in our reference samples. From this, we estimate a spin polarization for the Co/BP interface consistent with the *P*_Co/BP_ ≈ 85% previously observed in the Co/BP/Co device. Notably, if the actual Co/MgO spin polarization value were even larger than our assumed 40%, it would imply that in the symmetric junction the Co/BP spin polarization of the bottom interface is even higher than the already high value we report—further supporting the efficiency of spin injection through BP. This confirms the clear improvement in the signal obtained with the direct Co/BP/Co junction—with both Co/BP interfaces—compared to the Co/BP/MgO/Co hybrid junction, hence suggesting that the coupling between two Co electrodes through the BP flake leads to a much stronger spin-polarized current. This finding calls for further examination of the fundamental origin of this unusual result with semiconductors.

**Figure 3:**
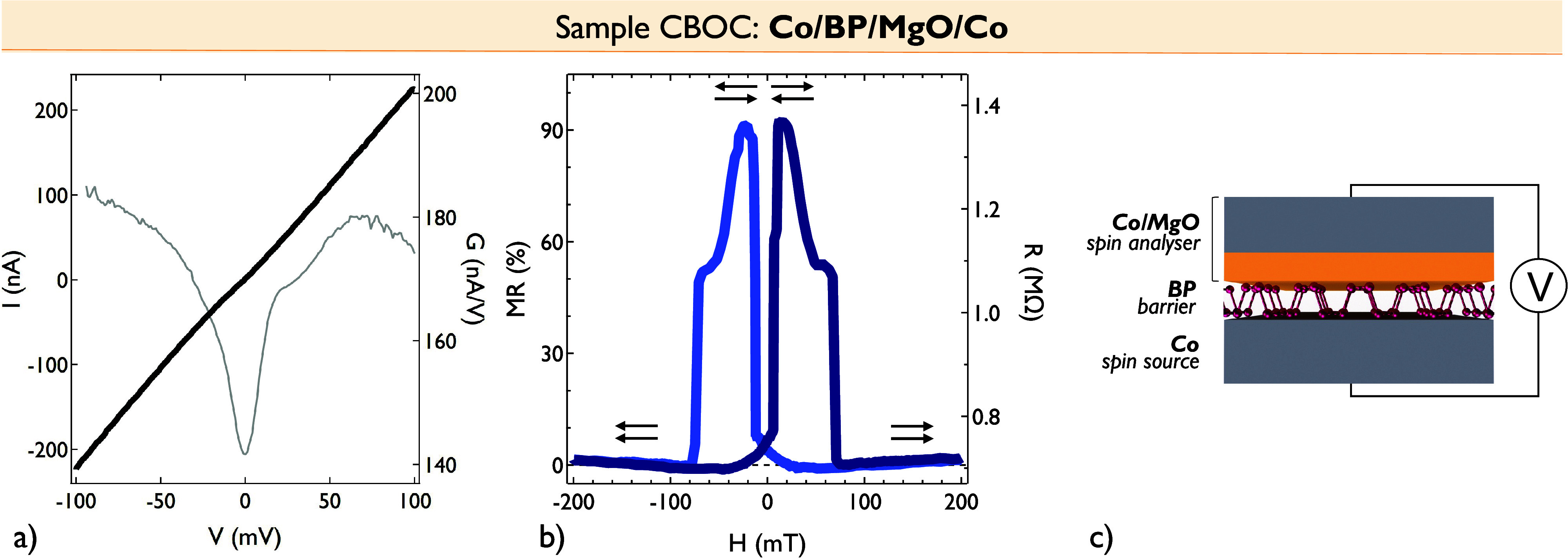
Magneto-transport measurements in a Co/BP/MgO/Co spin valve (Sample CBOC). (a) *IV* curve measured in the Co/BP/MgO/Co junction at 2 K that shows a nonlinear behavior (black curve). d*I*/d*V* curve (gray curve) that has been directly measured in the junction using an AC+DC lock-in system by applying a 10 mV AC signal. (b) MR signal (left axis) of 90% (with 3.6% error margin) and corresponding resistance R signal (right axis) measured in the Co/BP/MgO/Co junction at 2 K. (c) Schematic of the device.

To unveil the origin of the observed large MR signals stemming from BP, first-principles calculations were performed. Figure 4a depicts the Co/BP/Co junction considered in our theoretical analysis: a slab of six BP layers is sandwiched between two hcp ⟨0001⟩ cobalt electrodes. As already reported for other 2D semiconductors^27^ and in accordance with other works,^52^ BP is found to hybridize with the ferromagnetic electrode and become metallic close to the interface. As explained below, this hybridization leads to a differentiated selection of both spin channels. The semiconducting bandgap is recovered a few layers away from the surface. As an illustration, Figure 4b depicts the band structure of the BP projected on the outermost layer at the interface with the metal and on a bulk-like layer at the center of the stack. The corresponding spin-resolved projected density of states (DOS) and spin polarization are reported in Figure 4c. In all the layers, similar amplitudes for the DOS of spin ↑ and spin ↓ (spin directions are given with respect to the fixed magnetization of the bottom layer) are observed around the Fermi energy. This suggests that the origin of the large MR signal measured should not be ascribed to the bare polarization of the interfaces.

**Figure 4:**
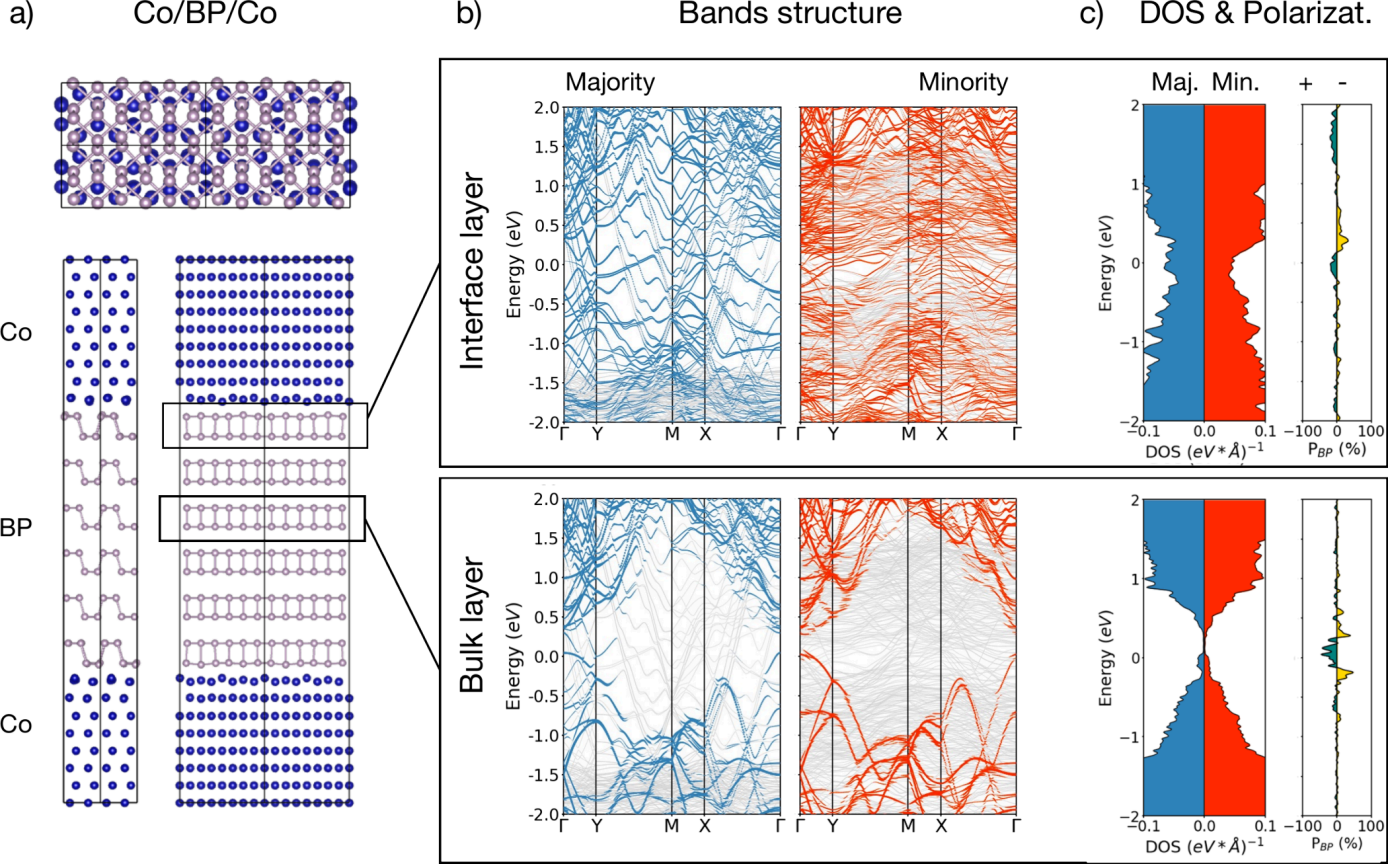
Electronic structure of the Co/BP multilayer/Co junction computed from first-principles. (a) Representation of the prototypical junction considered after relaxation of the internal coordinates. Cobalt and phosphor atoms are depicted as blue and brown balls, respectively. (b) Electronic band structure of the junction projected on the interface BP layer (upper panel) and bulk BP layer (lower panel). The full set of electronic bands (including the electrodes) is depicted in gray. The blue and red dots correspond to the projection of the majority and minority spin-eigenstates on the phosphor atoms of the selected layers. The size of the dots is proportional to the amplitude of the projection. (c) Density of states (left) and polarization (right) of selected layers of BP. The projected DOS of majority (minority) spin carriers is represented in blue (red). Positive (negative) spin-polarization is plotted in turquoise (gold). The Fermi level is taken as the energy reference. The spin polarization is calculated as *P* = (*N*_↑_ – *N*_↓_)/(*N*_↑_ + *N*_↓_), where *N*_↑(↓)_ is the spin dependent DOS at the Fermi level reported in the left panel.

To understand the spin-filtering mechanisms at play, we resort to nonequilibrium Green’s function (NEGF) transport calculations. The transmission functions computed at the Fermi level, spatially resolved in reciprocal space, are reported in Figure 5 for both the parallel (Figure 5a) and antiparallel (Figure 5b) magnetization configurations. One can see that the transmissions computed in the parallel configuration are higher and on a much broader reciprocal space surface than those in the antiparallel configuration, confirming the potential for high MR observed in the devices. One can also see that, as expected and owing to the symmetry of the junction, the transmission functions computed in the antiparallel configuration are spin-degenerate. This is not the case when the electrodes are magnetically aligned, leading to the key observation of our analysis. Interestingly, the spin ↑ and the spin ↓ transmission channels appear to follow separated paths in the reciprocal space, supporting the suggestion that the conventional spin polarization picture does not encompass the full mechanism here. One of the key figures is that the highest transmission channels are spin polarized with spin ↓ and are localized around Γ, while the spin ↑ channels mainly concentrate around X, at the edge of the Brillouin zone. This results in independent transmission paths for spin ↑ and spin ↓. As a consequence, the transport is strongly impeded in the antiparallel configuration, causing a very high antiparallel resistance state (R_AP_) and a large positive MR signal. This appears to be a unique combination of (i) spin ↑ and spin ↓ channels separately contributing at the X and Γ points, respectively, due to the Co/BP interfacial hybridization and (ii) a strong transmission attenuation at the X with respect to the Γ point as a function of thickness. Hence, the occurrence of strong spin filtering. To support this idea, Figure 5c reports the magneto-resistive signal ( $MR=\frac{T_{↑↑}+T_{↓↓}-{2T}_{↑↓}}{{2T}_{↑↓}}$ ) computed for different numbers of layers. A significant increase of the MR spin signal is predicted when increasing BP thickness from four to eight layers (L), fully in line with the description of those two orthogonal transmission paths taking over on interfacial spin polarization for thicker layers. A source of this filtering can be found in a peculiarity of BP. Indeed, as the number of layers is increased, the band gap at the Γ point is strongly reduced, while the gap at the X point is kept constant. This leads to different behaviors for the two channels. While a conventional exponential reduction of the transmission at the X point is expected as a function of thickness, at the Γ point, the transmission reduction is almost fully compensated by the gap reduction. Hence, increasing the number of layers leads to a stronger domination of the Γ (i.e., the spin ↓) channel, increasing the observed MR. This theoretical prediction is in very good agreement with our experimental observation of MR = 500% realized on ∼6 nm thick BP flake, corresponding to about 10 layers.^55^ Although our simulations were limited to eight layers due to the steep computational cost, the calculated MR for this thickness already approaches the experimental value, supporting the robustness of the theoretical model. Based on the observed trend, we would expect the MR to continue increasing for 10L, potentially reaching up to 700%. While this value would already be in good agreement with our experimental observations—given that real devices deviate from ideal conditions—practical factors such as spin depolarization are likely to moderate this increase at a certain point, bringing the theoretical prediction even closer to the measured MR.

**Figure 5:**
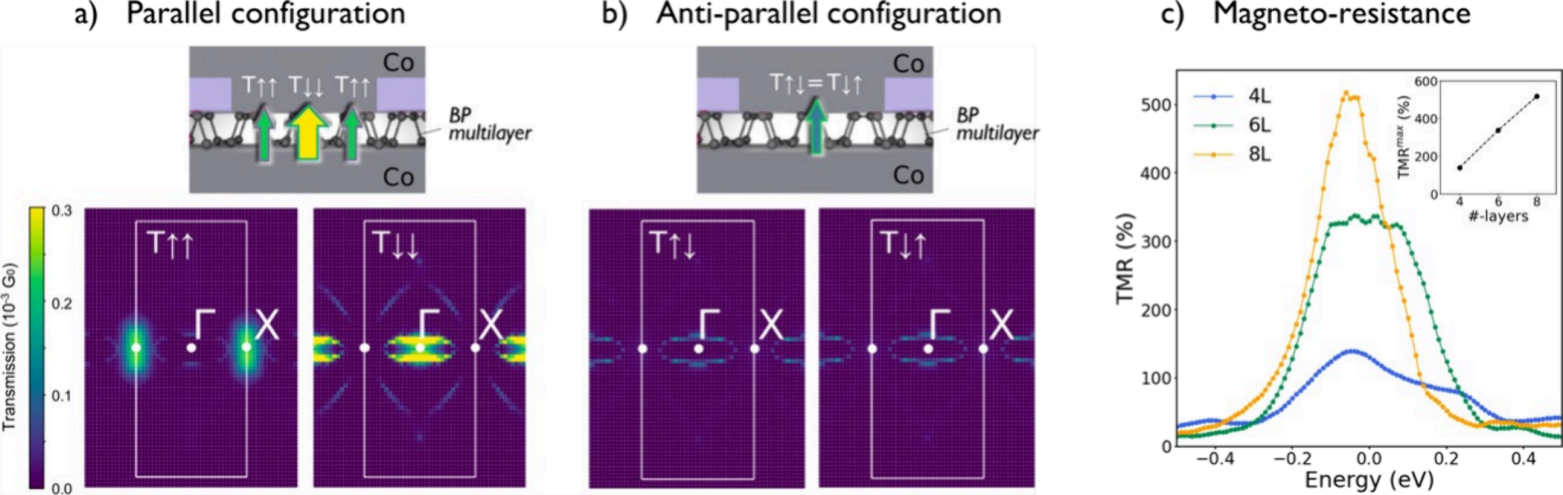
K-resolved transmission functions and magneto-resistance of the Co/BP/Co junction computed from non-equilibrium Green’s functions. Transmission functions of the parallel (a) and antiparallel (b) configurations at the Fermi energy. The first Brillouin zone associated with the computational supercell is represented by white lines. The transmission is expressed as a fraction of the quantum of conductance G_0_. (c) Magneto-resistance behavior computed for an increasing number of BP layers from 4L to 8L. A significant increase in MR signal is observed when flake thickness is increased due to larger spin filtering effects.

These results hence highlight the high potential of BP 2D material for spin valve devices. They unveil the remarkable origin of the large MR signal observed with BP layers due to the presence of completely different spin transport k-space paths for spin ↑ (at the X point) and spin ↓ (at the Γ point).

## Conclusion

In conclusion, we have demonstrated the successful fabrication of BP based vertical spin valves incorporating mechanically exfoliated flakes thanks to a specific fabrication process allowing BP passivation with oxide barriers (MgO and Al_2_O_3_) grown by ALD. Very high MR signals could be observed in these devices (up to 500%) corresponding to an equivalent 85% spin polarization of the current emitted from Co at the BP interface and corroborated by reference spin analyzer devices. This supersedes the highest value reported so far in 2D-based MTJs with ferromagnetic electrodes^56^ and an outstanding improvement compared to previous results reported in the literature on 2D semiconductor based MTJs.^19–29,46^ The nonequilibrium Green’s function transport analysis led to the unexpected conclusion that it is not the spin polarization at the Co/BP interface but the existence of two separate k-space channels for spin ↑ and spin ↓, related to the Co/BP hybridization at the interface, that lead to the extremely high MR signal observed in these devices.

Overall, these results support the outstanding potential of BP for spintronic devices. By demonstrating an exceptionally large spin filtering effect, combined with BP’s intrinsically long spin diffusion length (exceeding 6 μm, as reported by Avsar et al.^45^), our work highlights BP’s unique position among 2D semiconductors: it enables efficient spin injection, transport, and detection within a single material system. Such characteristics make it a compelling candidate to become a building block for spintronics post-CMOS visions toward all-spin logics architectures.

While this study provides a critical proof-of-concept to understand the underlying physics mechanism, it also opens multiple avenues for further exploration. As such, further optimization and focused studies are still required to fully leverage the BP potential and unravel the presented mechanism evolution for all possible device configurations. For example, a systematic investigation of temperature-dependent magnetoresistance will be crucial to assess the thermal robustness of the effect and understand its interplay with spin relaxation processes, as they are key considerations for real-world device operation.

Moreover, BP’s electronic structure—exhibiting momentum-dependent spin channel separation as revealed by our nonequilibrium Green’s function simulations, suggests that similar spin filtering phenomena could emerge in other members of the rich and diverse family of 2D semiconducting materials. Investigating these alternative systems could not only deepen our understanding of the underlying spin filtering mechanisms but also expand the design space for the next-generation 2D spintronics platform.

A community wide exploration is now required both from a theoretical point of view, treating more complex lateral structures, and an experimental point of view, providing the necessary technological development effort, including large scale growth, toward high-quality device optimization. We believe that the exposed promising proof-of-concept results will trigger such interest.

## Methods

### Bottom Spin Source Electrode Definition

The bottom Co electrode (20 nm) is deposited in a modified Plassys MP900S magnetron sputtering on a Si/SiO_2_ (90 nm) substrate in a high vacuum chamber with a base pressure of 10^–7^ mbar. Then, the sample is directly transferred using a dedicated vacuum suitcase into a glovebox where the H_2_O and O_2_ levels are kept below 1 ppm, thus avoiding sample surface oxidation. Inside the glovebox, a BP crystal (from HQ Graphene) is mechanically exfoliated using the scotch tape technique, and BP flakes are deposited on the Co surface. Afterward, again using the sealed transfer suitcase, the sample is transferred from the glovebox to the ALD growth chamber under an inert atmosphere. Hence, any exposure to air is avoided.

### Atomic Layer Deposition Growth

For hybrid Co/BP/MgO/Co junctions (Sample CBOC), a customized Beneq atomic layer deposition (ALD) growth system is used to grow a MgO layer of ∼2 nm covered with a protective layer of 10 nm of Al_2_O_3_. The Co/BP sample is directly transferred from the glovebox to the ALD growth chamber using a sealed transfer chamber which avoids air exposure. The growth chamber is heated at 100 °C. Before starting the growth, the sample is exposed to an ozone atomosphere for 2 min to promote adsorption of the molecule and subsequent nucleation. Then, it is exposed to 18 subsequent cycles of Mg precursor Mg­(EtCp)_2_, heated at 50 °C, and ozone. Successively, the growth chamber is heated to 300 °C and the sample is annealed inside for 2 h before growing the additional 10 nm Al_2_O_3_ protective layer through 100 subsequent cycles of ozone and tri­[methyl]­aluminum (TMA) precursor. Finally, the sample is left to cool down under vacuum overnight before being exposed to air. For symmetrical Co/BP/Co junctions (Sample CBC), the same ALD growth system is used to grow Al_2_O_3_ layers of 1 nm at the wetting limit. The Co/BP sample is transferred from the glovebox using the sealed suitcase to the ALD growth chamber. It is heated at 75 °C and exposed to 10 cycles of TMA and ozone with a growth rate of 1 Å/cycle. Finally, the sample is left to cool down under vacuum overnight before being exposed to air.

### HAADF SEM and TEM Measurements

A cross-sectional lamella was prepared using a FEI Helios 660 dual-beam microscope, which combines a field emission gun scanning electron microscope with an advanced focused Ga^+^ ion beam column operating at 30 kV. For TEM images, samples were examined using the JEOL NEOARM "MOSTRA" equipped with an annular dark field detector. The high-resolution Z-contrast imaging mode (STEM/HAADF) at 200 keV enabled us to achieve a resolution limit of below 100 pm

### Magnetic Tunnel Junction Fabrication

An AR-U-40.60 photoresist is spin-coated over the Co/BP/ALD passivation barrier sample with a final thickness of about 600 nm. Laser photolithography (Dilase 650 from Kloé) is used to open holes of 1–2 μm diameter over the selected flakes. Successively, for Sample CBOC, the 10 nm Al_2_O_3_ insulating layer is removed only inside the opened hole by chemical etching and contact is taken on the BP/MgO flake by sputtering deposition of the top Co (15 nm)/Au (80 nm) electrode. For Sample CBC, the top Co/Au electrode is deposited directly by sputtering over the structure. The junction cross-sectional area for the two devices will hence be in the order of some nm^2^ for the CBC nanocontact, while in the order of few μm^2^ for the CBOC device. Finally, a conductive epoxy drop is deposited over each junction and used as a mask during the ion beam etching process performed to electrically isolate the contacts.^27^ Samples are finally bonded into a chip and measured into a cryostat under an external in-plane magnetic field.

### Theoretical Calculations

The electronic structure calculations have been performed within the framework of density functional theory (DFT) as implemented within the SIESTA^57^ code with the vdW-DF functional of Dion et al.^58^ with the exchange modified by Klimeš et al.^59^ to describe the mean-field electronic interaction. Basis sets of numerical atomic orbitals (double-Zeta plus polarization) were used to expand the wave functions. Real-space quantities are represented on a regular grid characterized by an energy cutoff of 400 Ry. The ionic degrees of freedom of the two surface atomic layers of cobalt and the two first layers of BP were relaxed up to atomic forces lower than 0.01 eV/Å. The transmission functions have been computed within the framework of non-equilibirum Green’s functions (NEGFs).^60–62^ The transport calculations were performed self-consistently without applying any bias across the junction. Integration over the first Brillouin zone was performed on a regular 6 × 14 k-point grid for the self-consistent calculation of the electronic density with a Fermi–Dirac occupation function and an electronic temperature of 25 meV. While this does not correspond to the physical temperature of the system, such smearing is a standard technique used to facilitate convergence of integrals over the Brillouin zone and the self-consistent field loop. Importantly, this approach also demonstrates that the observed band filtering effect could persist up to room temperature, underscoring that this effect does not depend on fine features of the density of states, which would be smeared out at higher temperature, but rather on the robust, intrinsic band filtering mechanism we unveil in this system. Finally, a grid 5 times denser has been used for the non-self-consistent evaluation of the density of states and the transmission functions.

## ○

Email: marta.galbiati@uv.es
